# Tucum-do-Cerrado (*Bactris setosa* Mart.) May Promote Anti-Aging Effect by Upregulating SIRT1-Nrf2 Pathway and Attenuating Oxidative Stress and Inflammation

**DOI:** 10.3390/nu9111243

**Published:** 2017-11-14

**Authors:** Marcela de Sá Barreto da Cunha, Sandra Fernandes Arruda

**Affiliations:** 1 Postgraduate Program in Human Nutrition, Faculty of Health Sciences, Campus Universitário Darcy Ribeiro, Universidade de Brasília, Brasília 70910-900, Brazil; sandrafarruda@gmail.com; 2 Department of Nutrition, Faculty of Health Sciences, Campus Universitário Darcy Ribeiro, Universidade de Brasília, Brasília 70910-900, Brazil

**Keywords:** tucum-do-cerrado, phytochemicals, aging, sirtuins, Nrf2, oxidative stress, inflammation

## Abstract

Aging may be related to oxidative damage accumulation and a low-grade inflammation, both responses are modulated by iron and phytochemicals. This study investigated the effect of tucum-do-cerrado (*Bactris setosa* Mart.) consumption on the expression of sirtuins (SIRT 1 and 3) and senescence marker protein-30 (SMP30), and on the redox and inflammatory responses, in adult rats supplemented or not with dietary iron. Male Wistar rats were treated for 12 weeks with: control diet (CT); iron enriched-diet (+Fe); control diet + 15% tucum-do-cerrado (Tuc); or iron enriched-diet + 15% tucum-do-cerrado (Tuc + Fe). Iron supplementation (+Fe) increased liver, spleen and intestine iron levels, transferrin saturation, serum iron, serum TNF-α and IL-6 levels, hepatic carbonyl content and and superoxide dismutase (SOD) activity, hepatic Nrf2 protein and Nqo1 mRNA levels and decreased the renal Sirt1 mRNA levels in relation to CT group. Tucum-do-cerrado consumption (Tuc) increased hepatic SOD activity, Nrf2 and SIRT1 mRNA and protein contents, and Nqo1 mRNA levels, while it decreased the renal SOD activity compared with the CT diet. The consumption of tucum-do-cerrado associated with the iron-enriched diet (Tuc + Fe) increased the iron levels in tissues and serum transferrin saturation, compared to the CT diet, while promoting a decrease in hepatic carbonyl and renal malondialdehyde levels, marginally reducing serum IL-6 levels, and increasing hepatic SIRT1 protein content, renal Sirt1 and hepatic Nrf2 mRNA levels, compared to the +Fe group. None of the treatments altered Smp30 mRNA levels. The results suggest that tucum-do-cerrado consumption might promote an anti-aging effect by increasing SIRT1 expression, which may enhance Nrf2 mRNA and protein levels and its downstream pathway, which in turn decrease oxidative damage to proteins and the levels of inflammatory cytokines (IL-6 and TNF-α), induced by iron excess.

## 1. Introduction

In the last four decades, the number of older people has increased worldwide [1]. The development of aging is a multifactorial process [2,3]. According to some aging theories, the accumulation of molecular and cellular oxidative damages in all tissues throughout life and the disruption of immune response homeostasis, characterized by increased proinflammatory stimuli and decreased anti-inflammatory response, are determinant processes for development of chronic diseases and premature aging [2,4,5].

Although iron is an essential micronutrient for most organisms, its overload contributes to free radical generation and is closely correlated to the onset of some aging characteristics [6,7]. During the aging process, there is a greater availability of iron in its free form (“labile” iron pool) in tissues [8], which has been associated with an increased ability of the cell to produce reactive oxygen species (ROS), which in turn induces oxidative damage to biomolecules [9,10], increases inflammatory cytokines [11] and promotes the aging progression [9]. A previous study from our laboratory showed that dietary iron overload enhanced oxidative stress and the inflammatory process in 19-month-old rats [9], which may intensify the aging process.

On the other hand, phytochemicals modulate the expression of many genes and the activation of some proteins [12,13], which prevents the onset of chronic diseases and the aging progression [14,15]. Some phytochemicals induce the expression and activation of the transcription factor “nuclear factor erythroid 2-related factor 2” (Nrf2) [13,16], which upregulates the expression of antioxidant enzymes. This transcription factor also modulates the immune response, by downregulating the proinflammatory cytokine tumor necrosis-alpha (TNF-α) expression and the activity of the proinflammatory factor nuclear kappa B (NFkB) [13].

Recent studies have demonstrated that phytochemical compounds upregulate the NAD^+^-dependent histone/protein deacetylases sirtuins (SIRT1 and SIRT3) [17,18]. Sirtuins have been indicated as anti-aging proteins or longevity proteins [19,20] due to their ability to deacetylate and consequent activate the antioxidant enzymes and increase the reducing equivalents such as nicotinamide adenine dinucleotide phosphate (NADPH) and glutathione (GSH). It is hypothesized that the activation of SIRT1 promotes the deacetylation of Nrf2, improving the stability of this transcription factor [21,22]. The senescence marker protein-30 (SMP30), expressed mainly in the liver and kidney, is considered a biomarker of aging process, since its levels decrease with age [23,24]. It is suggested that SMP30 is able to suppress oxidative stress and consequently decrease NFkB levels, therefore, the decrease of SMP30 protein with age leads to an increase of oxidative damage and inflammatory response, which promotes aging [23,24].

The consumption of dietary sources of phytochemical compounds seems to attenuate oxidative damages and inflammatory status [12,15,25], promoting health benefits and preventing cell senescence [14,26,27]. Tucum-do-cerrado (*Bactris setosa* Mart.), a Brazilian savanna fruit, has a high content of different phytochemical compounds such as cyanidin-derivatives, epicatechin, catechin, piceatannol, quercetin, and all-*trans*-lutein, mainly found in the peel [25,27] and a high antioxidant potential in vitro and in vivo [28,29,30]; however, many other potential biological effects still need to be investigated. Therefore, we hypothesized that the dietary consumption of tucum-do-cerrado (*Bactris setosa* Mart.), attenuates the oxidative stress process and inflammatory response induced by iron supplementation, and consequently may modulate molecular markers associated with aging in adult rats.

To test this hypothesis, we investigated the iron status by assessing the levels of iron in the tissues (liver, spleen and intestine) and serum. The specific activity of the antioxidant enzymes, lipid peroxidation and protein carbonylation in the liver and kidney and the hepatic mRNA levels of nuclear factor erythroid derived 2 like 2 (Nfe2l2), NAD(P)H dehydrogenase quinone (Nqo1) and heme oxygenase-1 (Hmox1) were analyzed as markers of redox status. The inflammatory response was evaluated by the determination of interleukin 1 beta (Il1b) and tumor necrosis factor alpha (Tnfa) mRNA levels in the liver and the protein levels of IL-1β, IL-6 and TNF-α in the serum. The mRNA levels of sirtuin 1 (Sirt1), sirtuin 3 (Sirt3) and senescence marker protein 30 (Smp30) and the protein levels of SIRT1 and SIRT3 were determined as proteins sensitive to the aging process [17,18,23], in the liver and/or kidney [31,32], since these tissues are sensitive to redox balance and inflammation during the life.

## 2. Materials and Methods

### 2.1. Tucum-do-Cerrado Fruit Collection and Diet Preparation

Samples of tucum-do-cerrado fruit (*Bactris setosa* Mart.) were collected in the period of fructification at full maturity (March 2014), at Terezópolis de Goiás, 16°28′15.4″ S and 49°03′44.1″ W, Goiás, Brazil. The fruit identification was performed and a voucher specimen was deposited in the UB Herbarium of the University of Brasília, Brazil, with an identification number 124364. The permission to collect was issued by the *Instituto Brasileiro do Meio Ambiente e dos Recursos Naturais Renováveis* (IBAMA)/*Ministério do Meio Ambiente* (Authorization Number 9/2012, IBAMA/Ministério do Meio Ambiente). After the tucum-do-cerrado fruit collection, the seeds were removed and the pulp and the peel were stored at −80 °C until diet preparation. The diets were prepared monthly from the mixture of ingredients (Rhoster, Araçoiaba da Serra, SP, Brazil) following the proportion of the ingredients proposed by Reeves et al. (1993) [33]. In the diets that contained the tucum-do-cerrado, the pulp and the peel were homogenized in a blender with a small amount of water and subsequently homogenized to the other components of the diet. The diets were pelleted and stored at −20 °C.

### 2.2. Experimental Protocol

Twenty-four male Wistar rats (Granja GR, São Paulo, Brazil), 25 days-old (75.0 ± 6.6 g), were housed individually in stainless-steel cages, at a room temperature of 23 ± 2 °C, under a 12 h light cycle. Diets were offered only during the dark cycle, with free access to water. The Animal Care and Use Committee of the University of Brasília approved the experimental protocol (UnBDoc 20855/2014). After four weeks of acclimatization, until they reach adulthood, two month-old (248.9 ± 18.6 g), the animals were randomly allocated into four experimental groups (eight rats per group): the control group (CT) fed with the AIN-93G diet [33], that contained 35 mg of iron/kg of diet; the iron supplemented group (+Fe) received the AIN-93G diet containing 350 mg of iron kg of diet; the tucum-do-cerrado group (Tuc) were fed with the AIN-93G diet with 150 g of the edible parts (pulp and peel) of tucum-do-cerrado fruit/kg of diet added; and the tucum-do-cerrado + iron supplemented group (Tuc + Fe) received the AIN-93G diet enriched with 350 mg of iron and 150 g of the edible parts of the fruit/kg of diet. The food intake was recorded daily and the rats were weighted weekly. After 12 weeks of treatment, the five-month-old rats were anesthetized with isoflurane 3% and the blood was collected by cardiac puncture. The liver, spleen, intestine, and kidneys were excised, washed in saline 0.9%, rapidly frozen in liquid nitrogen and stored at −80 °C for further analysis.

### 2.3. Hematological Parameters

The hematological parameters: red blood cells (RBC), hemoglobin (Hb), hematocrit (Ht), mean corpuscular volume (MCV), platelets (PTL), and white blood cells (WBC) were determined in a cell counter (ABX Micros ESV 60, Horiba, Kyoto, Japan) using 1.5 mL of the blood. The cell counter was calibrated and the parameters were defined for rats.

### 2.4. Iron Status

#### 2.4.1. Total Iron Concentration in Tissues

The hepatic, splenic and intestinal total iron concentration was determined by inductively coupled plasma atomic emission spectrometry (Spectro, Kleve, Germany), as described by Baranowska et al. [34]. A calibration curve for iron was generated using standard mineral solutions (Titrizol, Merck, Darmstadt, Germany) in a concentration range of 0–10 ppm.

#### 2.4.2. Serum Iron Status

Serum iron, unsaturated iron binding capacity (UIBC), total iron binding capacity (TIBC), transferrin saturation, and transferrin levels were determined using commercial kits (Labtest, Lagoa Santa, Minas Gerais, Brazil), as described in the manufacturer’s assay protocol.

### 2.5. Oxidative Damage Markers and Antioxidant Capacity

#### 2.5.1. Carbonyl Protein and Lipid Peroxidation Levels

Protein oxidative damage was assessed by carbonyl protein levels in the liver and kidneys homogenates as described by Richert et al. [35]. The absorbance of the samples was measured at 376 nm (spectrophotometer Shimadzu-TCC 240A, Kyoto, Japan), and the carbonyl concentration in tissues was expressed as nmol carbonyl/mg of total protein, using the ε = 22,000 mM^−1^ cm^−1^. The lipid peroxidation levels in the liver and kidneys were determined by high performance liquid chromatography as proposed by Candan and Tuzmen [36]. The results were expressed as nmol malondialdehyde (MDA)/mg total protein. The total protein content in each homogenate was determined by the method of Hartree [37].

#### 2.5.2. Activity of Antioxidant Enzymes in the Liver and Kidney

The activity of catalase (CAT, EC 1.11.1.6), glutathione peroxidase (GPx, EC 1.11.1.9), glutathione reductase (GR, EC 1.6.4.2), and glutathione-S-transferase (GST, EC 2.5.1.18) was quantified in the liver and kidney as previously described [9]. The superoxide dismutase activity (SOD, EC 1.15.1.1) was determined using the protocol proposed by McCord [38]. The results were expressed as unit of enzyme/mg of total protein. The total protein content in each homogenate was determined by the method of Hartree [37].

### 2.6. Proinflammatory Cytokine Serum Levels

Serum levels of interleukin (IL)-1β, IL-6, and tumor necrosis-α (TNF-α) were measured using a commercially available enzyme-linked immunosorbent assay (ELISA) kit, according to the manufacturer’s assay protocol (Bender, MedSystems, Vienna, Austria).

### 2.7. Determination of Total RNA Extraction and Transcripts Levels

Total RNA extraction from liver and kidneys was performed using TRIzol reagent^TM^ (Invitrogen Inc., Burlington, ON, Canada) manufacturer’s assay protocol. Total RNA was used for complementary DNA (cDNA) synthesis using the High-Capacity cDNA Reverse Transcription Kit with RNase Inhibitor (Applied Biosystems, Foster City, CA, USA). The transcript levels of nuclear factor 2 related to factor 2 (Nfe2l2), NAD(P)H dehydrogenase quinone (Nqo1), heme oxygenase 1 (Hmox1), interleukin 1 beta (Il1b), tumor necrosis factor alpha (Tnfa), sirtuin 1 (Sirt1), sirtuin 3 (Sirt3), and senescence marker protein 30 (Smp30) were determined using real-time polymerase chain reaction (StepOne Real-Time PCR System, Applied Biosystems, Singapore). The mRNA levels of Nfe2l2, Nqo1, Hmox1, Il1b, and Tnfa were evaluated in the liver. Smp30 mRNA levels were determined in the kidney; Sirt1 and Sirt3 mRNA levels were assessed in the liver and kidney. The qRT-PCR reaction was performed using the Fast SYBR Green Master Mix 2x reagent, 2.0 μL of cDNA and 0.2 μmol/L (final concentration) of each primer, in a final volume of 10 μL. Primer sequences and GenBank accession number are presented in supplementary Table 1. The amplification specificity of each amplicon was verified by analyzing a melting curve. The comparative C_T_ method was used to quantify the abundance of target gene mRNA, and the results are presented as 2^−ΔΔC^_T_ [39]. All samples were assayed in triplicate and were normalized to the housekeeping gene β-actin (Actb).

### 2.8. Immunoblot Analysis

Samples of liver and kidney were homogenized in two volumes of 0.25 M sucrose, 15 mM Tris-HCl (pH 7.9), 15 mM NaCl, 60 mM KCl, 5 mM EDTA, 0.15 mM spermine, 0.5 mM spermidine, 0.1 mM phenylmethanesulfonyl fluoride (PMSF), 1.0 mM dithiothreitol, 1% protease inhibitor cocktail, and 1% phosphatase inhibitor cocktail (Sigma Aldrich, St. Louis, MO, USA), followed by sonication for one minute and centrifuged at 10,000× *g* for ten minutes at 4 °C (Universal Refrigerated Centrifuge Z 326 K, HERMLE, Gosheim, Germany). The supernatant was stored at −80 °C until the analysis. The total protein content in each extract was determined by the method of Hartree [37]. Protein was separated by SDS-PAGE (5–12%) [40], and transferred to a 0.45 mm polyvinylidene difloride (PVDF) membrane (ImmobilonH-P transfer membrane-IPVH00010-Millipore-Billerica, MA, USA), using a semi-dry transfer system (Trans-Blot^®^ SD Semi-Dry Transfer Cell, BIO-RAD, Hercules, CA, USA). Then, membranes were incubated with blocking buffer (TBS/T 1× 0.5% nonfat dry milk) for 2 h and then incubated with primary antibodies (diluted in 1× TBS/T + 0.5% nonfat dry milk) overnight at 4 °C. The dilutions used for each primary antibody are described in supplementary Table 2. After the incubation period, the membranes were washed three times with 1× TBS-T and incubated for 1 h with alkaline phosphatase-conjugated secondary antibody in a dilution of 1:1000 (# 7054-Anti-rabbit IgG, AP-linked Antibody, Cell Signaling, Danvers, MA, USA). The membranes were then washed three times with 1× TBS-T. The bands were visualized by BCIP^®^/NBT solution (B6404-Sigma Aldrich, St. Louis, MO, USA) and quantified using and ImageStudio Lite image analysis system (LI-COR Biosciences, Lincoln, NE, USA). The protein β-actin was used as constitutive protein. The data were expressed by the ratio between the intensities of the proteins of interest and β-actin, as arbitrary units (A.U.).

### 2.9. Statistical Analysis

Comparisons among Control group (CT) versus iron supplemented (+Fe), tucum-do-cerrado (Tuc) and tucum-do-cerrado + iron supplemented (Tuc + Fe) groups, and between +Fe versus Tuc + Fe groups were tested using One-Way Analysis of Variance (ANOVA) test with Bonferroni correction using SPSS version 17 software (SPSS Inc., Chicago, IL, USA). The level of statistical significance was set at *p* < 0.05. All values are expressed as the mean ± standard deviation (SD).

## 3. Results

### 3.1. Food Intake, Iron Intake, Weight Gain, and Hematological Parameters

The dietary iron supplementation (+Fe), the consumption of tucum-do-cerrado (Tuc), as well as the association of tucum-do-cerrado with the iron-enriched diet (Tuc + Fe) did not alter the food intake, the weight gain among and the hematological parameters among all of the treatment groups (Table 1 and Table 2). As expected, the iron intake was higher in the +Fe and Tuc + Fe groups compared with the CT group (*p* < 0.001 for both, Table 1), while no difference was observed between +Fe and Tuc + Fe groups. In the Tuc + Fe group the iron intake was higher than in the Tuc group (*p* < 0.001).

### 3.2. Iron Status

The treatment of rats with the iron-enriched diet (+Fe) promoted an increase in serum iron levels and transferrin saturation (TS), and a reduction in unbound iron-binding capacity (UIBC) compared with the CT diet (*p* = 0.031; *p* = 0.028, and *p* = 0.013, respectively, Table 3). Tucum-do-cerrado consumption did not change serum iron status. However, the consumption of the Tuc + Fe diet promoted a significant increase in transferrin saturation in relation to the CT group (*p* = 0.034; Table 3).

**Figure 1:**
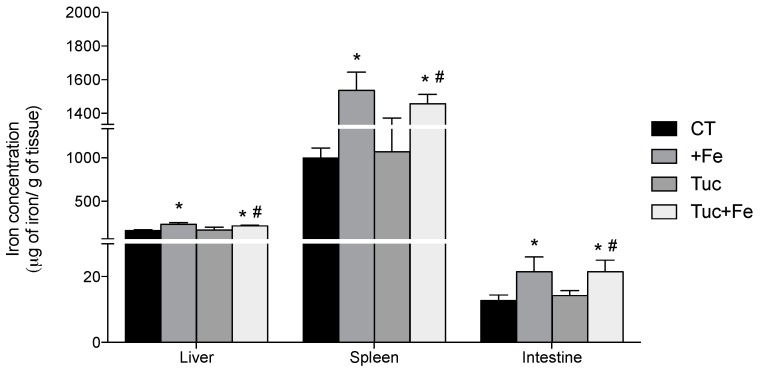
Effect of iron and/or tucum-do-cerrado intake in total iron concentration in the liver, spleen and intestine of adult rats treated for 12 weeks. Data correspond to average ± standard deviation (*n* = 6). * Statistical differences compared to CT group (*p* < 0.05). ^#^ Statistical differences compared to Tuc group (*p* < 0.05).

With respect to iron concentration in tissues, the dietary iron supplementation increased the iron levels in the liver, spleen and intestine of the +Fe (*p* = 0.001, 0.007 and < 0.001, respectively) and Tuc + Fe groups (*p* = 0.006, 0.016, 0.002, and 0.025, respectively) compared with the CT group (Figure 1). Tucum-do-cerrado consumption did not alter iron levels in the three studied tissues (Figure 1). The iron concentration in the liver, spleen and intestine of rats treated with Tuc + Fe diet was higher than the Tuc group (*p* = 0.020, 0.015 and 0.003, respectively).

### 3.3. Oxidative Damages and Antioxidant Capacity

**Figure 2:**
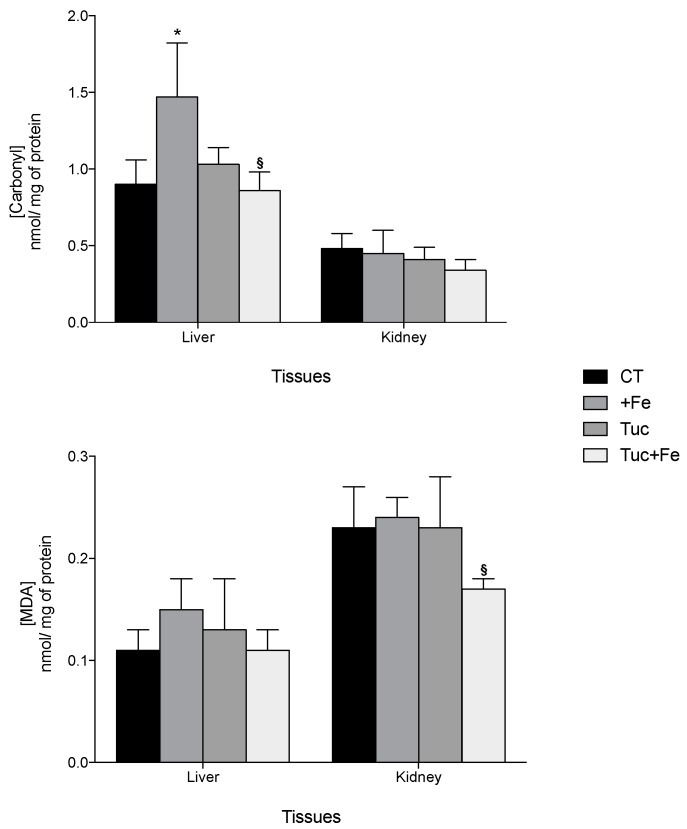
Effect of iron and/or tucum-do-cerrado intake in oxidative damage in the liver and in the kidney of adult rats treated for 12 weeks. Data correspond to average ± standard deviation (*n* = 6). * Statistical differences compared to CT group (*p* < 0.05). ^§^ Statistical differences compared to +Fe group (*p* < 0.05). MDA, malondialdehyde.

Regarding oxidative damages, the iron supplementation (+Fe) promoted an increase of hepatic carbonyl protein levels compared with the CT (*p* = 0.019; Figure 2). Although tucum-do-cerrado consumption did not change the levels of protein and lipid damage in all the studied tissues, the association of tucum-do-cerrado with the iron-enriched diet (Tuc + Fe) promoted a 1.7 and 1.4-fold decrease in liver carbonyl and kidney malondialdehyde (MDA) levels, respectively, compared with the +Fe group (*p* = 0.045 and 0.033, respectively; Figure 2). No difference was observed between Tuc + Fe and Tuc groups.

The antioxidant capacity was evaluated by determining the specific enzymatic activities of antioxidant enzymes in the liver and kidneys of the rats treated for 12 weeks with diets with added tucum-do-cerrado and/or iron-enriched (Table 4). Iron supplementation (+Fe) and tucum-do-cerrado consumption (Tuc) increased the activity of superoxide dismutase (SOD) in the liver in relation to the control group (*p* = 0.001 and 0.018, respectively). The association of iron supplementation with tucum-do-cerrado consumption (Tuc + Fe) marginally improved hepatic SOD activity (*p* = 0.067) and decreased glutathione reductase (GR) activity compared with the CT group (*p* = 0.039). Moreover, the hepatic glutathione peroxidase (GPx) activity was increased in the Tuc + Fe group compared to +Fe group (*p* = 0.048).

In the kidney, tucum-do-cerrado comsumption (Tuc) as well as its association with iron supplementation (Tuc + Fe) deacresed SOD activity compared with the CT (*p* = 0.005 and < 0.001, respectively). Catalase (CAT) and superoxide dismutase (SOD) activities were decreased in the Tuc + Fe group compared with +Fe group (*p* = 0.029 and *p* < 0.001, Table 4), while the glutathione reductase activity (GR) was lower than the Tuc group (*p* = 0.007).

### 3.4. Nrf2-Related Pathway

**Figure 3:**
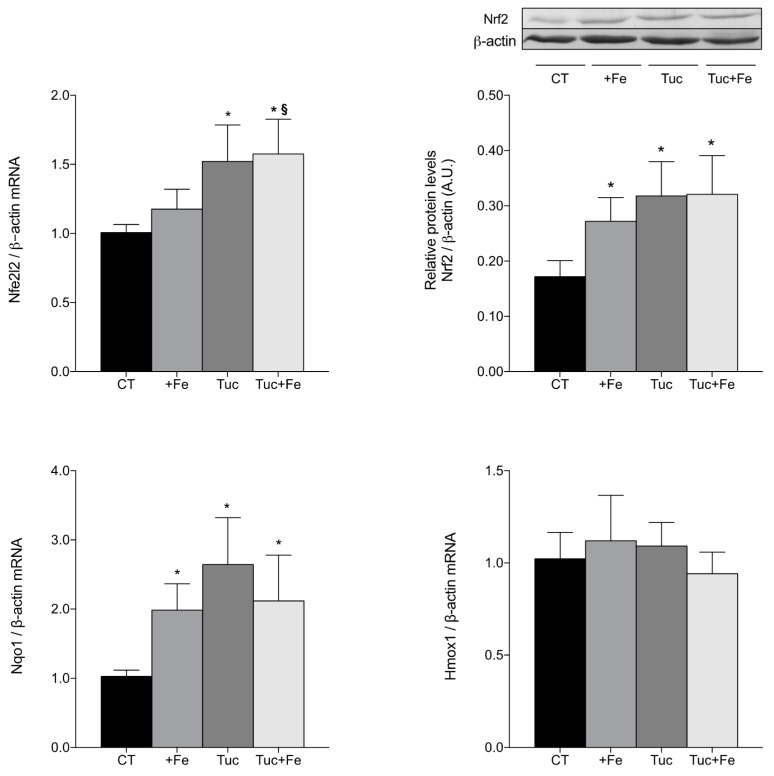
Hepatic mRNA levels quantification of nuclear factor erythroid 2-related factor 2 gene (Nfe2l2) and protein (Nrf2), NAD(P)H dehydrogenase quinone 1 (Nqo1) and heme-oxygenase 1 (Hmox1) mRNA levels of adult rats treated for 12 weeks. Data correspond to average ± standard deviation (*n* = 6). * Statistical differences compared to CT group (*p* < 0.05). ^§^ Statistical difference compared to +Fe group (*p* < 0.05).

Considering that the nuclear factor erythroid 2-related factor 2 (Nrf2)-related pathway modulates the expression of the antioxidant enzymes, we evaluated the transcript levels of Nfe2l2 (gene that encodes Nrf2 protein) and the protein levels of Nrf2 in the liver (Figure 3). The iron supplementation (+Fe) significantly increased Nrf2 protein in the liver, in relation to the CT group (*p* = 0.043; Figure 3), although the Nfe2l2 mRNA levels was not altered, in relation to the CT group. The consumption of tucum-do-cerrado increased the hepatic Nrf2 mRNA and protein levels compared with the CT group (*p* = 0.002, for both); similar results were observed comparing Tuc + Fe to the CT group (*p* < 0.001 for Nfe2l2 mRNA; *p* = 0.001 for Nrf2 protein levels). The Tuc + Fe group also showed higher mRNA levels of hepatic Nfe2l2 in relation to the +Fe group (*p* = 0.013); however, no difference was observed in Nrf2 protein levels between these groups.

In order to investigate whether Nrf2 is in its active form, the mRNA levels of two Nrf2-target genes, NAD(P)H dehydrogenase quinone 1 (Nqo1) and heme-oxygenase 1 (Hmox1), were assessed and the results are presented in Figure 3. All the treatment groups (+Fe, Tuc, and Tuc + Fe) showed an increase of Nqo1 mRNA levels in the liver compared with the CT group (*p* = 0.024, 0.000 and 0.009, respectively); however, no difference was obtained in hepatic Hmox1 mRNA levels among all groups.

### 3.5. Inflammatory Markers

**Figure 4:**
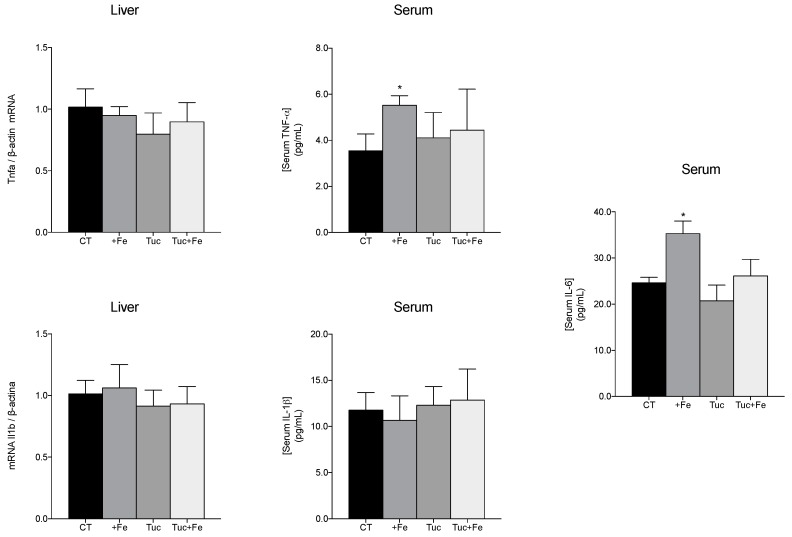
Quantification of the levels of hepatic interleukin-1b mRNA (Il1b) and serum protein (IL-1β), hepatic tumor necrosis alpha mRNA (Tnfa) and protein TNF-α and IL-6 in serum of adult rats treated for 12 weeks. Data correspond to average ± standard deviation (*n* = 6). * Statistical differences compared to CT group (*p* < 0.05).

To evaluate the effect of tucum-do-cerrado on the inflammatory response, the hepatic mRNA levels, and the serum levels of some proinflammatory cytokines were determined (Figure 4). The consumption of the iron-enriched diet (+Fe) promoted an increase in the tumor necrosis factor-alpha (TNF-α) and interleukin-6 (IL-6) serum levels, in relation to the CT group (*p* = 0.024 and *p* = 0.028, respectively); however, no change was obtained in the hepatic mRNA levels of Tnfa. The consumption of tucum-do-cerrado (Tuc) did not alter the levels of the studied inflammatory markers; however, the association of tucum-do-cerrado to the iron-enriched diet (Tuc + Fe) marginally decreased the serum IL-6 levels compared with the +Fe group (*p* = 0.055; Figure 4). No difference was observed in serum protein and hepatic mRNA levels of IL-1β among all treatment groups (Figure 4).

### 3.6. Aging-Related Markers

**Figure 5:**
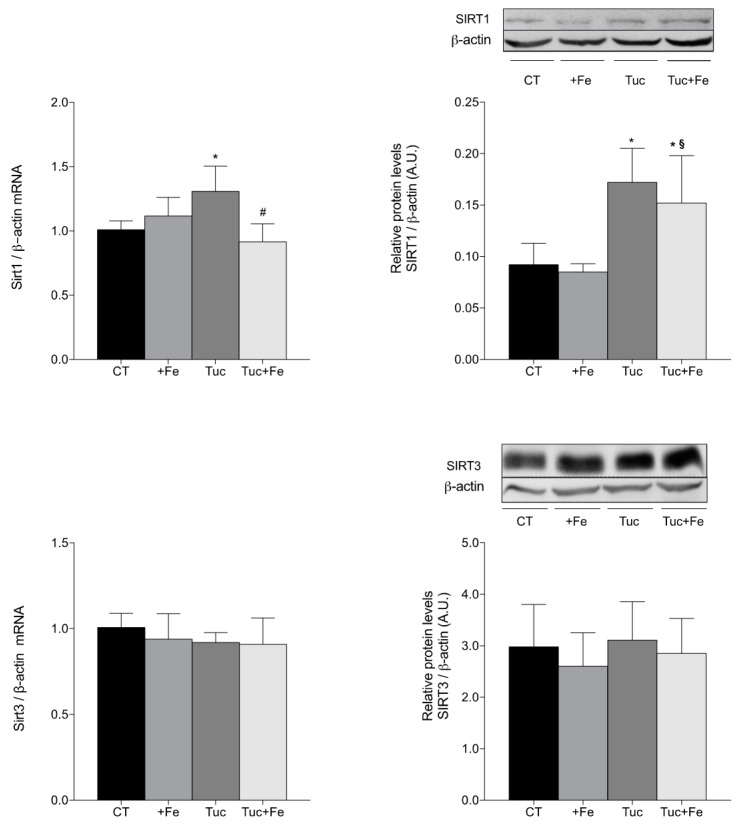
Hepatic mRNA quantification of Sirt1 and Sirt3 and protein levels determination of SIRT1 and SIRT3 in liver of adult rats treated for 12 weeks. Data correspond to average ± standard deviation (*n* = 6). * Statistical differences compared to CT group (*p* < 0.05). ^§^ Statistical difference compared to +Fe group (*p* < 0.05). ^#^ Statistical difference compared to Tuc group (*p* < 0.05).

**Figure 6:**
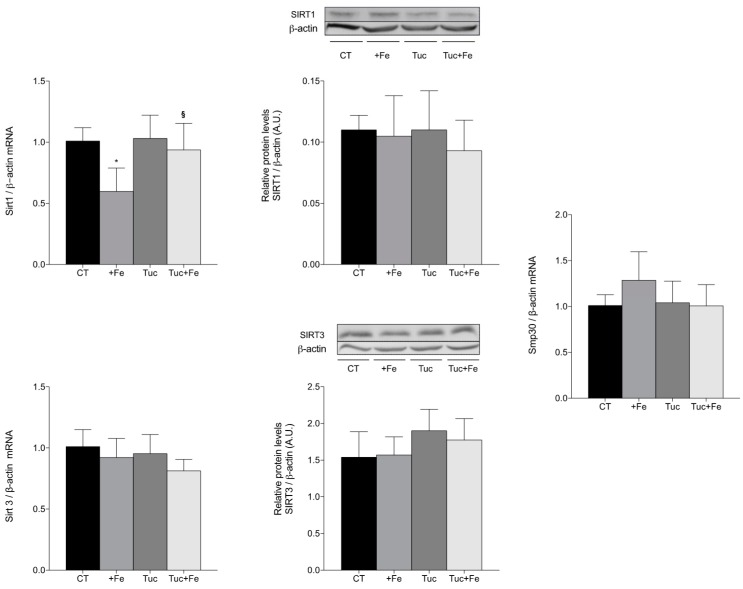
Renal mRNA quantification of Sirt1, Sirt3 and Smp30 and protein levels determination of SIRT1 and SIRT3 in kidney of adult rats treated for 12 weeks. Data correspond to average ± standard deviation (*n* = 6). * Statistical differences compared to CT group (*p* < 0.05). ^§^ Statistical difference compared to +Fe group (*p* < 0.05).

The hepatic and renal SIRT1 and SIRT3 mRNA and protein levels as well as the renal Smp30 mRNA levels were determined in order to evaluate the effect of tucum-do-cerrado on the aging process (Figure 5 and Figure 6). The iron-enriched diet (+Fe) did not alter the aging-related proteins among the different treatments, in the liver (Figure 5). The rats treated with tucum-do-cerrado diet (Tuc) showed higher mRNA and protein levels of SIRT1 in relation to the CT group (*p* = 0.012 and *p* = 0.003, respectively). The Tuc + Fe group showed an increase only in the protein levels of hepatic SIRT1 in relation to the CT group (*p* = 0.030) and +Fe group (*p* = 0.009). A significant decrease of Sirt1 mRNA levels were observed in Tuc + Fe group compared with Tuc group (*p* = 0.001). There were no significant differences in the hepatic SIRT3 mRNA and protein levels among the different treatments (Figure 5).

In the kidney (Figure 6), the iron supplementation (+Fe) promoted a significant decrease in Sirt1 mRNA levels compared with the CT group (*p* = 0.005), while the tucum-do-cerrado consumption (Tuc group) did not alter the mRNA and protein levels of SIRT1 compared with the control diet. The addition of tucum-do-cerrado to the iron-enriched diet (Tuc + Fe) increased the kidney mRNA levels of Sirt1 compared with the +Fe group (*p* = 0.025; Figure 6), although no significant difference was observed for protein concentration. No differences were observed in the mRNA levels of SIRT3 and Smp30, and in the SIRT3 protein levels in the kidney, among the different treatments (Figure 6).

## 4. Discussion

In the current study, the administration of a diet containing 350 mg Fe/kg during 12 weeks resulted in an iron overload, because serum iron, transferrin saturation, and iron concentration in the liver, spleen, and intestine were increased in the +Fe group compared with the control group, whereas the unbound iron-binding capacity (UIBC) was significantly diminished in the +Fe group. In higher concentrations, a greater proportion of iron remains weakly bounded, and this iron pool can catalyze free radical generation, triggering oxidative stress and inflammation [41,42], which are two processes that are closely related to aging progression and lifespan limitation [43]. In the present study, the higher protein oxidative damage associated with the increase of superoxide dismutase activity (SOD), observed in the liver of the +Fe group compared with the CT group, suggests that iron excess increases free radical generation, and that the cell improves antioxidant defense in order to avoid oxidative stress condition. Similar to our findings, other studies have shown that iron-induced overload increases oxidative damage and specific activities of the antioxidant enzymes [44,45]. The authors suggest that this increase may be related to a feedback response to a mild oxidative stress that triggers the activation of nuclear factor related to factor 2 (Nrf2), which in turn increases cell antioxidant defenses to control the iron-induced ROS generation [44]. Therefore, in the present study, the higher Nrf2 protein level, associated with the higher NAD(P)H dehydrogenase quinone 1 mRNA (Nqo1) levels and hepatic SOD activity observed in the liver of the iron-enriched group, suggests that the mild oxidative stress caused by iron supplementation was able to induce and activate the Nrf2 protein in an attempt to increase the antioxidant capacity and consequently control damage caused by iron excess.

Although the addition of tucum-do-cerrado to the control diet did not alter oxidative damages in the tissues analyzed, in the iron overload condition, tucum-do-cerrado consumption (Tuc + Fe group) protected the liver and kidneys from oxidative damages catalyzed by iron. The Tuc + Fe group showed lower hepatic carbonyl and renal malondialdehyde content in relation to the +Fe group, which suggests an antioxidant potential of tucum-do-cerrado in the presence of a potent oxidant. Furthermore, the upregulation of hepatic Nfe2l2 mRNA and protein levels promoted by tucum-do-cerrado consumption (Tuc and Tuc + Fe groups) seems to be modulated by the interaction of phytochemicals with antioxidant responsive elements, present in the Nfr2 gene (Nfe2l2). The literature shows that the phytochemical, quercetin, is able to induce hepatic Nrf2 at transcriptional and posttranscriptional levels, as well as by its dissociation from the repressor Keap1 [46]. Although we evaluated only the total hepatic Nrf2 protein content, our results also suggest that tucum-do-cerrado consumption activates the Nrf2 protein, due to the upregulation of Nqo1 mRNA levels (Figure 3), a Nrf2 target-gene [16], and the marginal increase of hepatic SOD activity of both the Tuc and Tuc + Fe groups in relation to control. Unexpectedly, no difference was observed in heme oxygenase-1 (Hmox1) mRNA levels, another Nrf2 target gene.

In the kidney, the lower enzymatic activities of SOD and catalase (CAT) in the Tuc and Tuc + Fe rats did not increase oxidative damages to biomolecules (Figure 2 and Table 4), instead the Tuc + Fe group showed a decrease in lipid peroxidation in the kidney compared with the +Fe group (+Fe; Figure 2 and Table 4). Considering that phytochemicals and its metabolites present a heterogeneous distribution among tissues and that kidneys are identified as one of the tissues that most accumulates these compounds [47,48,49], it is possible that a greater deposition of tucum-do-cerrado phytochemicals in the kidney of the Tuc and Tuc + Fe rats may have led to increased non-enzymatic antioxidant capacity in this tissue sparing the endogenous enzymatic antioxidant defenses.

In agreement with literature data [9,41], the consumption of the iron-enriched diet for 12 weeks promoted an increase in the proinflammatory state, as the +Fe group had increased tissue iron levels, serum tumor necrosis factor alpha (TNF-α) and interleukin (IL)-6. Despite the high phytochemical content of tucum-do-cerrado, no significant alteration was found in the levels of the serum proinflammatory cytokines (TNF-α, IL-1β, and IL-6) due to tucum-do-cerrado consumption when compared with the CT group. However, in the presence of iron excess, a pro-oxidant and proinflammatory agent, the consumption of tucum-do-cerrado (Tuc + Fe group) promoted a marginal decrease of serum IL-6 levels in relation to the +Fe group (*p* = 0.055), and even though the serum TNF-α levels of the Tuc + Fe group was not different from the +Fe group, it was similar to the control group. Therefore, these results suggest an anti-inflammatory activity of tucum-do-cerrado in the presence of a stressor agent. Similar results were observed in an iron-overloaded mice model, in which iron supplementation increased serum IL-6 and TNF-α levels, while the treatment of these mice with resveratrol significantly decreased these inflammatory biomarkers [41]. Niu et al. [18] demonstrated that treatment of aged rats (112 weeks-old) with the polyphenol epigallocatechin gallate (EGCG) significantly reduced oxidative stress markers, TNF-α, and IL-6 serum levels and increased lifespan by 8 to 12 weeks compared with non-treated animals of the same age [18].

Corroborating to the hypothesis that iron overload promotes the oxidative and inflammatory responses and that these responses are closely related to the aging progression, in the present study the iron supplementation (+Fe group) promoted a reduction of Sirt1 mRNA levels in the kidney compared with the CT group, which suggests that iron excess may contribute to aging progression. However, no difference was observed in hepatic Sirt1 mRNA levels, even though the iron concentration in the liver of the +Fe group was higher than the control group. In contrast to our findings, Das et al. [50] observed that the chronic intraperitoneal injection of iron dextran reduced SIRT1 protein levels in the liver. These apparent contradictory findings may be attributed to the distinct routes of iron administration, since Das et al. [50] used intraperitoneal injection of dextran-iron, while in the present study the iron was dietetically administrated.

**Figure 7:**
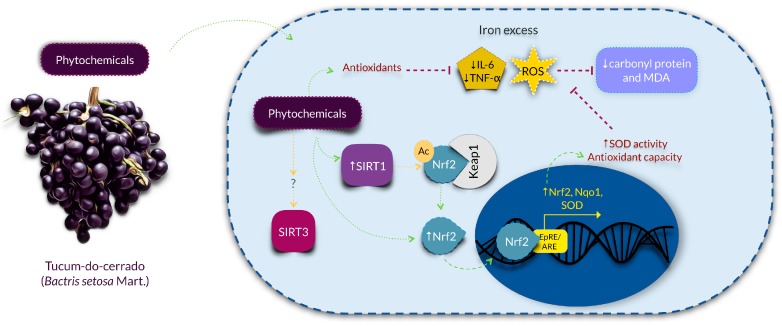
Proposed mechanism of action of the phytochemical compounds of tucum-do-cerrado in the prevention of aging, by the upregulation SIRT1-Nrf2 pathway, attenuating oxidative stress and inflammation, in the liver. Tucum-do-cerrado is a source of phytochemical compounds, which might enter the cells and act directly as anti-inflammatory molecules and/or reducing agents, promoting the upregulation of the longevity protein SIRT1. The upregulation of SIRT1 may causes the deacetylation of Nrf2, making it stable and active. Nrf2 migrates to the nucleus and induces antioxidant enzymes and its own transcription, rising the cell antioxidant capacity. Then, by reduction of the ROS generation, the oxidative damages to protein and lipids are lowered and the inflammatory response is attenuated. The effect of tucum-do-cerrado in SIRT3 remains to be elucidated. Ac: acetylation; IL-6: interleukin-6; Keap1: Kelch-like ECH-associated protein 1; MDA: malondialdehyde; Nqo1: NAD(P)H dehydrogenase quinone 1; Nrf2: nuclear factor erythroid 2-related factor 2; ROS: reactive oxygen species; SIRT1: sirtuin 1; SIRT3: sirtuin 3; SOD: superoxide dismutase; TNF-α: tumor necrosis alpha.

According to our hypothesis, tucum-do-cerrado consumption (Tuc group) upregulated hepatic Sirt1 mRNA expression and SIRT1 protein concentration, and this response was observed even in the presence of iron supplementation (Tuc + Fe group). Corroborating our results, Niu et al. [18] demonstrated that rats treated throughout life (about 108 weeks) with epigallocatechin galatte (EGCG, 25 mg/kg/day) showed an increase in SIRT1 levels in liver and kidney, and a reduction in oxidative stress and inflammatory markers compared with the control group. Das et al. [50] also observed that the administration of resveratrol (320 mg/kg diet/day, for 14 weeks) increased hepatic SIRT1 protein concentration and activity in iron-overloaded mice. An in vitro study demonstrated that the treatment of lung cells of senescent mice with oligonol, a polyphenol present in lychee and in grape seeds, promoted an attenuation of ROS generation, increased the levels of SIRT1 protein, and activated the SIRT1-AMPK pathway [51]. The authors suggested that the activation of this pathway might increase the autophagy process, an important anti-aging mechanism, which is normally diminished with age [51]. Therefore, as SIRT1 is downregulated by ROS generation [19], we hypothesized that the improvement of SIRT1 expression by tucum-do-cerrado consumption is modulated by the increase of the cellular reducing potential. A recent study observed that the resveratrol glycoside, polydatin, increased SIRT1 expression, which promoted the deacetylation and consequent activation of Nrf2, resulting in the upregulation of Nrf2 target genes and higher antioxidant capacity [21]. Thus, considering that SIRT1 is a longevity protein [18,52], we suggest that tucum-do-cerrado may promote healthier aging by increasing SIRT1 mRNA and protein levels, which in turn activates Nrf2-related pathway and, thereafter, attenuates the oxidative and inflammatory responses. This proposed anti-aging mechanism is presented in Figure 7.

SIRT3 is another histone-deacetylase protein, whose expression is modulated by redox response and its content is positively linked to an increase of life expectancy [17,18,53]. In the present study, although the iron-supplemented group (+Fe) showed higher protein oxidative damage and the tucum-do-cerrado treated groups (Tuc and Tuc + Fe) received a higher content of polyphenols, no significant differences were observed in SIRT3 mRNA and protein levels in the liver and kidney, among the treatments. Similar to the present study, Asseburg et al. [18] observed no difference in hepatic and cerebral Sirt3 mRNA levels in aged mice (18 to 21 months-old) treated with grape peel extract (200 mg/kg/day for 3 weeks), while Sirt1 mRNA levels were increased compared with the control aged group.

A higher antioxidant capacity and lower inflammatory response are associated with an increase of SMP30 expression [24]; however, as far as we know, no studies have associated changes in SMP30 levels with phytochemical treatment. In the present study, no difference was observed in renal Smp30 mRNA levels among all treatments, despite the increase of the oxidative damages observed in the +Fe group and the increased in the antioxidant potential (lower levels of oxidative damage, increased SOD activity and Nf2l2 mRNA, and protein upregulation and activation) promoted by tucum-do-cerrado consumption (Tuc and Tuc + Fe) and the marginal reduction in proinflammatory cytokines levels when tucum-do-cerrado was combined with the iron-enriched diet (Table 4 and Figure 2, Figure 3 and Figure 4). An earlier study of our group showed that adult rats (19 months) treated with an iron-enriched diet had lower levels of renal Smp30 mRNA in relation to young rats (2 months); however, renal Smp30 mRNA levels were not altered in adult rats treated with iron-enriched diet compared with those treated with control diet [9]. Therefore, in the present study the changes in the antioxidant/oxidant responses promoted by the different treatments during 12 weeks seems to be insufficient to modulate SMP30 expression in the kidney.

## 5. Conclusions

The results suggest that continuing tucum-do-cerrado consumption might promote an anti-aging effect by enhancing SIRT1, which may activate Nrf2 mRNA and protein levels and its downstream pathway, in the liver, which in turn attenuates oxidative damage to proteins and the levels of inflammatory cytokines (IL-6 and TNF-α), induced by iron excess.

## Figures and Tables

**Table 1:** Effect of iron and/or tucum-do-cerrado intake in the body weight gain, dietary intake and dietary iron intake of adult rats treated for 12 weeks.

	CT	+Fe	Tuc	Tuc + Fe
Body weight gain (g)	218.7 ± 29.2	223.8 ± 26.8	195.3 ± 31.4	197.2 ± 39.8
Dietary intake (g/12 weeks)	1558.6 ± 106.8	1642.0 ± 89.6	1534.7 ± 73.7	1604.7 ± 188.9
Dietary iron intake (mg/12 weeks)	38.5 ± 2.6	352.4 ± 19.2 *	44.0 ± 2.1	347.7 ± 40.9 *^,#^

CT, group treated with control diet AIN-93G; +Fe, group treated with AIN-93G diet containing 350 mg of iron/kg of diet; Tuc, AIN-93G diet added of 15% tucum-do-cerrado; Tuc + Fe, group treated AIN-93G diet containing 350 mg of iron/kg of diet and added of 15% tucum-do-cerrado. Data correspond to average ± standard deviation (*n* = 6). * Statistical differences compared to CT group (*p* < 0.05). ^#^ Statistical differences compared to Tuc group (*p* < 0.05).

**Table 2:** Effect of iron and/or tucum-do-cerrado intake in the hematological parameters of adult rats treated for 12 weeks.

	CT	+Fe	Tuc	Tuc + Fe
RBC (×10^6^/μL)	7.95 ± 0.63	7.84 ± 0.60	7.77 ± 0.60	7.29 ± 0.48
Hb (g/dL)	15.07 ± 0.94	14.77 ± 0.31	15.29 ± 0.95	15.19 ± 1.10
HCT (%)	42.88 ± 1.03	41.28 ± 1.35	42.50 ± 1.50	40.50 ± 1.66
PTL (×10^3^/μL)	590.08 ± 56.53	544.08 ± 85.26	524.17 ± 16.68	589.10 ± 48.06
WBC (×10^3^/μL)	4.86 ± 1.35	4.36 ± 0.69	4.18 ± 0.89	4.85 ± 0.88

CT, group treated with control diet AIN-93G; +Fe, group treated with AIN-93G diet containing 350 mg of iron/kg of diet; Tuc, AIN-93G diet added of 15% tucum-do-cerrado; Tuc + Fe, group treated AIN-93G diet containing 350 mg of iron/kg of diet and added of 15% tucum-do-cerrado. RBC: red blood cells; Hb: hemoglobin; HCT: hematocrit; PTL: platelets; WBC: white blood cells. Data correspond to average ± standard deviation (*n* = 6).

**Table 3:** Effect of iron and/or tucum-do-cerrado in the serum iron parameters of adult rats treated for 12 weeks.

	CT	+Fe	Tuc	Tuc + Fe
Serum iron (μg/dL)	107.68 ± 5.85	202.46 ± 78.42 *	142.17 ± 32.98	177.60 ± 42.02
UIBC (μg/dL)	286.18 ± 59.85	199.64 ± 41.21 *	256.15 ± 20.93	260.98 ± 37.59
TIBC (μg/dL)	405.67 ± 48.94	380.64 ± 74.93	398.32 ± 45.08	438.58 ± 30.92
TS (%)	26.77 ± 3.76	40.80 ± 9.27 *	37.13 ± 3.16	40.40 ± 8.50 *
Tf (mg/dL)	283.97 ± 34.26	266.45 ± 52.45	278.82 ± 31.55	307.01 ± 21.64

CT, group treated with control diet AIN-93G; +Fe, group treated with AIN-93G diet containing 350 mg of iron/kg of diet; Tuc, AIN-93G diet added of 15% tucum-do-cerrado; Tuc + Fe, group treated AIN-93G diet containing 350 mg of iron/kg of diet and added of 15% tucum-do-cerrado. UIBC: unbound iron-binding capacity; TIBC: total iron-binding capacity; TS: transferrin saturation; Tf: transferrin. Data correspond to average ± standard deviation (*n* = 6). * Statistical differences compared to CT group.

**Table 4:** Effect of iron and/or tucum-do-cerrado intake in antioxidant enzymes activity in the liver and kidney of adult rats treated for 12 weeks.

Enzyme	Tissue	CT	+Fe	Tuc	Tuc + Fe
CAT	Liver	208.89 ± 24.96	167.53 ± 40.80	266.28 ± 51.32	223.87 ± 28.11
	Kidney	103.85 ± 13.03	116.95 ± 15.46	91.05 ± 11.57	91.16 ± 8.94 ^§^
GPx	Liver	1.07 ± 0.11	0.74 ± 0.03	1.33 ± 0.29	1.13 ± 0.18 ^§^
	Kidney	0.64 ± 0.14	0.63 ± 0.10	0.54 ± 0.10	0.57 ± 0.11
SOD	Liver	17.23 ± 1.16	37.83 ± 4.49 *	29.99 ± 7.89 *	28.17 ± 3.74
	Kidney	19.44 ± 1.76	21.99 ± 3.14	13.13 ± 1.30 *	10.67 ± 1.12 *^,§^
GR	Liver	38.37 ± 10.84	36.35 ± 6.67	42.48 ± 5.45	22.81 ± 3.80 *
	Kidney	133. 68 ± 30.21	139.90 ± 17.72	108.33 ± 21.24	97.00 ± 6.36
GST	Liver	340.09 ± 76.14	428.47 ± 74.26	454.54 ± 93.64	427.16 ± 73.22
	Kidney	124.16 ± 17.62	139.10 ± 24.83	125.56 ± 23.67	118.42 ± 12.22

CT, group treated with control diet AIN-93G; +Fe, group treated with AIN-93G diet containing 350 mg of iron/kg of diet; Tuc, AIN-93G diet added of 15% tucum-do-cerrado; Tuc + Fe, group treated AIN-93G diet containing 350 mg of iron/kg of diet and added of 15% tucum-do-cerrado. CAT, catalase; GPx, glutathione peroxidase; SOD, superoxide dismutase; GR, glutathione reductase; GST, glutathione S transferase. Data correspond to average ± standard deviation (*n* = 6). * Statistical differences compared to CT group; ^§^ Statistical differences compared to +Fe group (*p* < 0.05).

## Supplementary Materials

The following are available online at www.mdpi.com/2072-6643/9/11/1243/s1, Table S1: Primer sequences used for Nfe2l2, Nqo1, Hmox1, Il1b, Tnfa, Smp30, Sirt1, Sirt3 and Actb real-time PCR assays, Table S2: Dilutions and companies of primary antibodies used for protein immunoblotting.

## References

[1] WHO (2017). Global Health and Aging—What Are the Public Health Implications of Global Ageing.

[2] Monti D., Ostan R., Borelli V., Castellani G., Franceschi C. (2017). Inflammaging and human longevity in the omics era. Mech. Ageing Dev..

[3] Rattan S.I. (2006). Theories of biological aging: Genes, proteins, and free radicals. Free Radic. Res..

[4] Edrey Y.H., Salmon A.B. (2014). Revisiting an age-old question regarding oxidative stress. Free Radic. Biol. Med..

[5] Schottker B., Saum K.U., Jansen E.H., Boffetta P., Trichopoulou A., Holleczek B., Dieffenbach A.K., Brenner H. (2015). Oxidative stress markers and all-cause mortality at older age: A population-based cohort study. J. Gerontol. Ser. A Biol. Sci. Med. Sci..

[6] Becerril-Ortega J., Bordji K., Freret T., Rush T., Buisson A. (2014). Iron overload accelerates neuronal amyloid-beta production and cognitive impairment in transgenic mice model of Alzheimer’s disease. Neurobiol. Aging.

[7] Fernandez-Real J.M., Manco M. (2014). Effects of iron overload on chronic metabolic diseases. Lancet Diabetes Endocrinol..

[8] Doulias P.T., Vlachou C., Boudouri C., Kanavaros P., Siamopoulos K.C., Galaris D. (2008). Flow cytometric estimation of ’labile iron pool’ in human white blood cells reveals a positive association with ageing. Free Radic. Res..

[9] Arruda L.F., Arruda S.F., Campos N.A., de Valencia F.F., Siqueira E.M. (2013). Dietary iron concentration may influence aging process by altering oxidative stress in tissues of adult rats. PLoS ONE.

[10] Xu J., Hwang J.C., Lees H.A., Wohlgemuth S.E., Knutson M.D., Judge A.R., Dupont-Versteegden E.E., Marzetti E., Leeuwenburgh C. (2012). Long-term perturbation of muscle iron homeostasis following hindlimb suspension in old rats is associated with high levels of oxidative stress and impaired recovery from atrophy. Exp. Gerontol..

[11] Bowie A., O’Neill L.A. (2000). Oxidative stress and nuclear factor-kappab activation: A reassessment of the evidence in the light of recent discoveries. Biochem. Pharmacol..

[12] Si H., Liu D. (2014). Dietary antiaging phytochemicals and mechanisms associated with prolonged survival. J. Nutr. Biochem..

[13] Qin S., Hou D.X. (2016). Multiple regulations of Keap1/Nrf2 system by dietary phytochemicals. Mol. Nutr. Food Res..

[14] Porquet D., Casadesus G., Bayod S., Vicente A., Canudas A.M., Vilaplana J., Pelegri C., Sanfeliu C., Camins A., Pallas M. (2013). Dietary resveratrol prevents Alzheimer’s markers and increases life span in SAMP8. Age.

[15] Del Bo C., Martini D., Porrini M., Klimis-Zacas D., Riso P. (2015). Berries and oxidative stress markers: An overview of human intervention studies. Food Funct..

[16] Stefanson A.L., Bakovic M. (2014). Dietary regulation of Keap1/Nrf2/are pathway: Focus on plant-derived compounds and trace minerals. Nutrients.

[17] Asseburg H., Schafer C., Muller M., Hagl S., Pohland M., Berressem D., Borchiellini M., Plank C., Eckert G.P. (2016). Effects of grape skin extract on age-related mitochondrial dysfunction, memory and life span in C57BL/6J mice. Neuromol. Med..

[18] Niu Y., Na L., Feng R., Gong L., Zhao Y., Li Q., Li Y., Sun C. (2013). The phytochemical, EGCG, extends lifespan by reducing liver and kidney function damage and improving age-associated inflammation and oxidative stress in healthy rats. Aging Cell.

[19] Yuan Y., Cruzat V.F., Newsholme P., Cheng J., Chen Y., Lu Y. (2016). Regulation of SIRT1 in aging: Roles in mitochondrial function and biogenesis. Mech. Ageing Dev..

[20] Ansari A., Rahman M.S., Saha S.K., Saikot F.K., Deep A., Kim K.H. (2017). Function of the SIRT3 mitochondrial deacetylase in cellular physiology, cancer, and neurodegenerative disease. Aging Cell.

[21] Huang K., Chen C., Hao J., Huang J., Wang S., Liu P., Huang H. (2015). Polydatin promotes Nrf2-are anti-oxidative pathway through activating SIRT1 to resist ages-induced upregulation of fibronetin and transforming growth factor-beta1 in rat glomerular messangial cells. Mol. Cell. Endocrinol..

[22] Li S., Zhao G., Chen L., Ding Y., Lian J., Hong G., Lu Z. (2016). Resveratrol protects mice from paraquat-induced lung injury: The important role of SIRT1 and Nrf2 antioxidant pathways. Mol. Med. Rep..

[23] Kondo Y., Masutomi H., Noda Y., Ozawa Y., Takahashi K., Handa S., Maruyama N., Shimizu T., Ishigami A. (2014). Senescence marker protein-30/superoxide dismutase 1 double knockout mice exhibit increased oxidative stress and hepatic steatosis. FEBS Open Bio.

[24] Jung K.J., Lee E.K., Kim S.J., Song C.W., Maruyama N., Ishigami A., Kim N.D., Im D.S., Yu B.P., Chung H.Y. (2015). Anti-inflammatory activity of SMP30 modulates NF-kappab through protein tyrosine kinase/phosphatase balance. J. Mol. Med..

[25] Joseph S.V., Edirisinghe I., Burton-Freeman B.M. (2016). Fruit polyphenols: A review of anti-inflammatory effects in humans. Crit. Rev. Food Sci. Nutr..

[26] Oyebode O., Gordon-Dseagu V., Walker A., Mindell J.S. (2014). Fruit and vegetable consumption and all-cause, cancer and CVD mortality: Analysis of health survey for england data. J. Epidemiol. Community Health.

[27] Valenzano D.R., Terzibasi E., Genade T., Cattaneo A., Domenici L., Cellerino A. (2006). Resveratrol prolongs lifespan and retards the onset of age-related markers in a short-lived vertebrate. Curr. Biol..

[28] Boeing J.S., Ribeiro D., Chiste R.C., Visentainer J.V., Costa V.M., Freitas M., Fernandes E. (2017). Chemical characterization and protective effect of the bactris setosa Mart. fruit against oxidative/nitrosative stress. Food Chem..

[29] Rosa F.R., Arruda A.F., Siqueira E.M., Arruda S.F. (2016). Phytochemical compounds and antioxidant capacity of tucum-do-cerrado (bactris setosa mart), Brazil’s native fruit. Nutrients.

[30] Fustinoni-Reis A.M., Arruda S.F., Dourado L.P., da Cunha M.S., Siqueira E.M. (2016). Tucum-do-cerrado (*Bactris setosa* Mart.) consumption modulates iron homeostasis and prevents iron-induced oxidative stress in the rat liver. Nutrients.

[31] Zhang Y., Chen X., Yang L., Zu Y., Lu Q. (2015). Effects of rosmarinic acid on liver and kidney antioxidant enzymes, lipid peroxidation and tissue ultrastructure in aging mice. Food Funct..

[32] Feng Y., Yu Y.H., Wang S.T., Ren J., Camer D., Hua Y.Z., Zhang Q., Huang J., Xue D.L., Zhang X.F. (2016). Chlorogenic acid protects d-galactose-induced liver and kidney injury via antioxidation and anti-inflammation effects in mice. Pharm. Biol..

[33] Reeves P.G., Nielsen F.H., Fahey G.C. (1993). AIN-93 purified diets for laboratory rodents: Final report of the american institute of nutrition ad hoc writing committee on the reformulation of the AIN-76A rodent diet. J. Nutr..

[34] Baranowska I., Czernicki K., Aleksandrowicz R. (1995). The analysis of lead, cadmium, zinc, copper and nickel content in human bones from the upper silesian industrial district. Sci. Total Environ..

[35] Richert S., Wehr N.B., Stadtman E.R., Levine R.L. (2002). Assessment of skin carbonyl content as a noninvasive measure of biological age. Arch. Biochem. Biophys..

[36] Candan N., Tuzmen N. (2008). Very rapid quantification of malondialdehyde (MDA) in rat brain exposed to lead, aluminium and phenolic antioxidants by high-performance liquid chromatography-fluorescence detection. Neurotoxicology.

[37] Hartree E.F. (1972). Determination of protein: A modification of the lowry method that gives a linear photometric response. Anal. Biochem..

[38] McCord J.M. (2001). Analysis of superoxide dismutase activity. Current Protocols in Toxicology.

[39] Livak K.J., Schmittgen T.D. (2001). Analysis of relative gene expression data using real-time quantitative PCR and the 2(-delta delta c_T_) method. Methods.

[40] Azevedo M.O., Felipe M.S.S., Brígido M.M., Maranhão A.Q., De-Souza M.T. (2010). Técnicas Básicas Em Biologia Molecular.

[41] Zhao L., Wang Y., Wang Z., Xu Z., Zhang Q., Yin M. (2015). Effects of dietary resveratrol on excess-iron-induced bone loss via antioxidative character. J. Nutr. Biochem..

[42] Chaudhuri D., Ghate N.B., Panja S., Das A., Mandal N. (2015). Wild edible fruit of *prunus nepalensis* ser. (steud), a potential source of antioxidants, ameliorates iron overload-induced hepatotoxicity and liver fibrosis in mice. PLoS ONE.

[43] Salminen A., Ojala J., Kaarniranta K., Kauppinen A. (2012). Mitochondrial dysfunction and oxidative stress activate inflammasomes: Impact on the aging process and age-related diseases. Cell. Mol. Life Sci..

[44] Piloni N.E., Perazzo J.C., Fernandez V., Videla L.A., Puntarulo S. (2016). Sub-chronic iron overload triggers oxidative stress development in rat brain: Implications for cell protection. Biometals.

[45] Silva-Gomes S., Santos A.G., Caldas C., Silva C.M., Neves J.V., Lopes J., Carneiro F., Rodrigues P.N., Duarte T.L. (2014). Transcription factor NRF2 protects mice against dietary iron-induced liver injury by preventing hepatocytic cell death. J. Hepatol..

[46] Tanigawa S., Fujii M., Hou D.X. (2007). Action of Nrf2 and Keap1 in ARE-mediated NQO1 expression by quercetin. Free Radic. Biol. Med..

[47] Serra A., Macia A., Romero M.P., Angles N., Morello J.R., Motilva M.J. (2011). Distribution of procyanidins and their metabolites in rat plasma and tissues after an acute intake of hazelnut extract. Food Funct..

[48] Kawabata K., Mukai R., Ishisaka A. (2015). Quercetin and related polyphenols: New insights and implications for their bioactivity and bioavailability. Food Funct..

[49] Kim Y.J., Lee D.H., Ahn J., Chung W.J., Jang Y.J., Seong K.S., Moon J.H., Ha T.Y., Jung C.H. (2015). Pharmacokinetics, tissue distribution, and anti-lipogenic/adipogenic effects of allyl-isothiocyanate metabolites. PLoS ONE.

[50] Das S.K., DesAulniers J., Dyck J.R., Kassiri Z., Oudit G.Y. (2016). Resveratrol mediates therapeutic hepatic effects in acquired and genetic murine models of iron-overload. Liver Int..

[51] Park S.K., Seong R.K., Kim J.A., Son S.J., Kim Y., Yokozawa T., Shin O.S. (2016). Oligonol promotes anti-aging pathways via modulation of SIRT1-AMPK-autophagy pathway. Nutr. Res. Pract..

[52] Giblin W., Skinner M.E., Lombard D.B. (2014). Sirtuins: Guardians of mammalian healthspan. Trends Genet..

[53] Rose G., Dato S., Altomare K., Bellizzi D., Garasto S., Greco V., Passarino G., Feraco E., Mari V., Barbi C. (2003). Variability of the SIRT3 gene, human silent information regulator Sir2 homologue, and survivorship in the elderly. Exp. Gerontol..

