# Supplementary material for: Tucum-do-Cerrado (Bactris setosa Mart.) May Promote Anti-Aging Effect by Upregulating SIRT1-Nrf2 Pathway and Attenuating Oxidative Stress and Inflammation
Source: Nutrients. 2017 Nov 14;9(11):1243. doi: 10.3390/nu9111243 (PMC5707715; doi:10.3390/nu9111243)
Supplement: Supplementary file 1 [file nutrients-09-01243-s001.pdf]

Supplementary Table 1. Primer sequences used for Nfe2l2, Nqo1, Hmox1, Il1b, Tnfa, Smp30, Sirt1, Sirt3 and Actb real-time PCR assays.

Gene	Primers sequences (5′- 3′)	GenBank accession number
**Nfe2l2**	GAGACGGCCATGACTGAT (forward)<br>GTGAGGGGATCGATGAGTAA (reverse)	NM_031789.2
**Nqo1**	CAGCGGCTCCATGTACT (forward)<br>GACCTGGAAGCCACAGAAG (reverso)	NM_017000.3
**Hmox1**	ATCGTGCTCGCATGAAC (forward)<br>CAGCTCCTCAAACAGCTCAA (reverse)	NM_012580
**Il1b**	CACCTCTCAAGCAGAGCACAG (forward)<br>GGGTTCCATGGTGAAGTCAAC (reverse)	NW_047658
**Tnfa**	AAATGGGCTCCCTCTCATCAGTTC (forward)<br>GTCGTAGCAAACCACCAAGCAGA (reverse)	X66539
**Smp30**	AGGCATCAAAGTGTCTGCTGTTT (forward)<br>GACTGTCGAAGTGCCACTGAACT (reverse)	X69021
**Sirt1**	CTGTTTCCTGTGGGATACCTGACT (forward)<br>ATCGAACATGGCTTGAGGATCT (reverse)	XM_003751934
**Sirt3**	CCCGCTGCCCTGTCTGT (forward)<br>CTCCCCAAAGAACACAATGTCA (reverse)	NM_001106313
**Actb**	GTCGTACCACTGGCATTGTG (forward)<br>CTCTCAGCTGTGGTGGTGAA (reverse)	NM_031144

Supplementary Table 2. Dilutions and companies of primary antibodies used for protein immunoblotting.

Protein	Dilution	Code/Company
SIRT1	1:1000	#9475 / Cell Signaling Technology
SIRT3	1:1000	#5490 / Cell Signaling Technology
Nrf2	1:200	sc-13032 / Santa Cruz Biotechnology
β-actin	1:1000	#4967S / Cell Signaling Technology
